# Sex differences in mitochondrial gene expression during viral myocarditis

**DOI:** 10.21203/rs.3.rs-3716881/v1

**Published:** 2023-12-19

**Authors:** Damian Di Florio, David Gorelov, Elizabeth McCabe, Danielle Beetler, Katie Shapiro, Katelyn Bruno, Isha Chekuri, Angita Jain, Emily Whelan, Gary Salomon, Sami Khatib, Natalie Bonvie-Hill, Presley Giresi, Varsini Balamurugan, Gabriel Weigel, Jessica Fliess, Ashley Darakjian, Brandy Edenfield, Christian Kocsis, Christopher McLeod, Leslie Cooper, Etienne Audet-Walsh, Michael Coronado, Jon Sin, DeLisa Fairweather

**Affiliations:** Mayo Clinic in Florida; Mayo Clinic in Florida; Mayo Clinic in Florida; Mayo Clinic in Florida; Mayo Clinic in Florida; University of Florida; Mayo Clinic in Florida; Mayo Clinic in Florida; Mayo Clinic in Florida; Mayo Clinic in Florida; Mayo Clinic in Florida; Mayo Clinic in Florida; Mayo Clinic in Florida; Mayo Clinic in Florida; Mayo Clinic in Florida; Mayo Clinic in Florida; Mayo Clinic in Florida; Mayo Clinic in Florida; Mayo Clinic in Florida; Mayo Clinic in Florida; Mayo Clinic in Florida; Université Laval: Universite Laval; Cytokinetics Inc; The University of Alabama; Mayo Clinic Florida

**Keywords:** inflammation, innate immunity, coxsackievirus B3, peroxisome proliferator-activated receptor gamma coactivator 1/PGC1a, nuclear respiratory factor 1/NRF1, estrogen-related receptor alpha estrogen/ERRa, autoimmune disease, interleukin-1 beta, mitochondria

## Abstract

**Background:**

Myocarditis is an inflammation of the heart muscle most often caused by an immune response to viral infections. Sex differences in the immune response during myocarditis have been well described but upstream mechanisms in the heart that might influence sex differences in disease are not completely understood.

**Methods:**

Male and female BALB/c wild type mice received an intraperitoneal injection of heart-passaged coxsackievirus B3 (CVB3) or vehicle control. Bulk-tissue RNA-sequencing was conducted to better understand sex differences in CVB3 myocarditis. We performed enrichment analysis to understand sex differences in the transcriptional landscape of myocarditis and identify candidate transcription factors that might drive sex differences in myocarditis.

**Results:**

The hearts of male and female mice with myocarditis were significantly enriched for pathways related to an innate and adaptive immune response compared to uninfected controls. When comparing females to males with myocarditis, males were enriched for inflammatory pathways and gene changes that suggested worse mitochondrial transcriptional support (e.g., mitochondrial electron transport genes). In contrast, females were enriched for pathways related to mitochondrial respiration and bioenergetics, which were confirmed by higher transcript levels of master regulators of mitochondrial function including peroxisome proliferator-activated receptor gamma coactivator 1 (PGC1α), nuclear respiratory factor 1 (NRF1) and estrogen-related receptor alpha (ERRα). TRANSFAC analysis identified ERRa as a transcription factor that may mediate sex differences in mitochondrial function during myocarditis.

**Conclusions:**

Master regulators of mitochondrial function were elevated in females with myocarditis compared to males and may promote sex differences in mitochondrial respiratory transcript expression during viral myocarditis resulting in less severe myocarditis in females following viral infection.

## Plain English Summary

Many viruses can infect the heart. Immune cells go to the heart to get rid of the virus but sometimes this response can damage the heart. When immune cells go to the heart it is called “myocarditis,” which means ‘myo’ for muscle and ‘carditis’ for heart inflammation. More men get myocarditis after viral infections than women. When we study viral infection in mice as a model of human disease, we see the same sex differences as in humans. To know why this happens, we gave mice a viral infection and looked at how their hearts changed. We found that a special gene acts like a like a *light switch* to turn on the genes that help cell energy factories, called “mitochondria.” Females used this light switch to turn on the genes that help mitochondria, but males did not and turned most of these genes off. We learned that the light switch works better in females compared to males, so it may play an important role in protecting female’s hearts during myocarditis. More researched is needed to better understand how this light switch works. Understanding how to turn on mitochondria genes in the heart could help doctors also do this in men after a viral infection to prevent myocarditis and save patients’ lives.

## Background

Myocarditis is an inflammation of the myocardium, or muscle tissue of the heart, and a leading cause of sudden cardiac death in persons under 50 years of age [1, 2]. The Global Burden of Disease (GBD) study from 2019 reported 1.8 million cases of myocarditis world-wide [2]. A 2014 Swedish study reported myocarditis at an incidence of 8.6 people per 100,000 [3]. Several epidemiological studies estimated at least a 15-fold increased incidence of myocarditis/perimyocarditis from severe acute respiratory syndrome coronavirus-2 (SARS-CoV-2) infection during the coronavirus disease 2019 (COVID-19) pandemic [4, 5]. Prior to the pandemic, coxsackievirus B3 (CVB3) was the leading suspected cause of myocarditis in the United States. Myocarditis can progress to dilated cardiomyopathy (DCM) in susceptible individuals and in mouse models of viral and autoimmune myocarditis [6–8]. In this model, upregulated profibrotic remodeling genes during acute myocarditis (day 10 post infection/pi) lead to the development of fibrosis and ventricular dilation during DCM (day 35 pi and onwards) [9, 10]. Chronic heart failure during DCM leads to heart transplants in a significant proportion of patients [11, 12]. A lack of disease-specific therapies aside from heart failure medications provides impetus for identification of novel biomarkers and therapeutic targets with the goal of earlier detection and more targeted treatment.

The incidence and severity of myocarditis is greater in cis-males (referred to hereafter as males) than cis-females (referred to hereafter as females) in humans and mouse models [8]. The GBD study reported a mortality rate in patients with myocarditis aged 35–39 of 4.4 per 100,000 in women and 6.1 per 100,000 in men, indicating that more men die of myocarditis than women worldwide [2]. We previously reported a sex ratio of 3.5 males to 1 female among patients with biopsy confirmed myocarditis [1]. Men are more likely to develop cardiac fibrosis and progress to DCM after myocarditis compared to women [10, 11, 13]. We, and others, previously reported that testosterone promotes a proinflammatory and profibrotic response in an autoimmune model of CVB3 myocarditis while estrogen is cardioprotective [1, 10, 14, 15]. The goal of this study was to better understand sex differences in CVB3 myocarditis using bulk-tissue RNA sequencing (RNAseq). We found major sex differences in transcriptional programming related to cardiac mitochondrial biogenesis.

## Results

### Myocardial inflammation is increased in males compared to females

**Figure 1:**
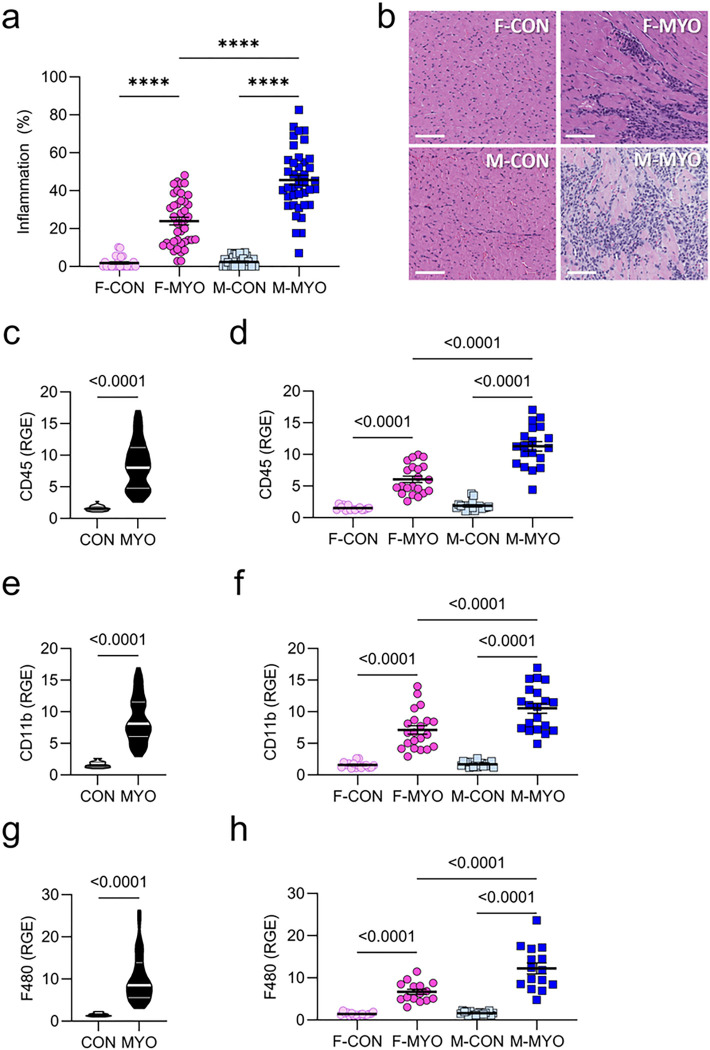
Myocardial inflammation is increased in males compared to females **a,** Myocarditis severity (% inflammation) between female controls (F-CON, *n*= 27), females with myocarditis (F-MYO, *n* = 41), male controls (M-CON, *n*= 30), and males with myocarditis (M-MYO, *n* = 40); **b,** representative heart sections (scale bars = 80μm); **c-h,** relative gene expression (RGE) for controls (CON, *n* = 31–34) versus mice with myocarditis (MYO, *n* = 32–41) and for F-CON (*n* = 15–16), F-MYO (*n* = 15–21), M-CON (*n* = 18), and M-MYO (*n* = 15–20).

We first examined inflammation in males versus females and uninfected PBS vehicle controls to confirm sex differences and to select samples for RNA sequencing. As we have shown previously [16], males in our autoimmune CVB3 model develop significantly more inflammation than females (*p* = 0.0001) according to histological assessment whereas vehicle controls did not develop myocarditis (Figure 1a). Representative examples of histology for each group are shown in Figure 1b. We confirmed major immune cell types in the heart of males vs. females with myocarditis compared to controls using qRT-PCR. We found total immune cells (CD45, *p* < 0.0001), complement 3 activated myeloid cells (CD11b, *p* < 0.0001), and macrophages (F4/80, *p* < 0.0001) were increased in males with myocarditis compared to females with myocarditis (Figure 1c–h), as expected [16]. Thus, males have greater cardiac inflammation during autoimmune CVB3 myocarditis than females.

### Females upregulate while males downregulate gene pathways related to mitochondrial homeostasis during myocarditis

**Figure 2:**
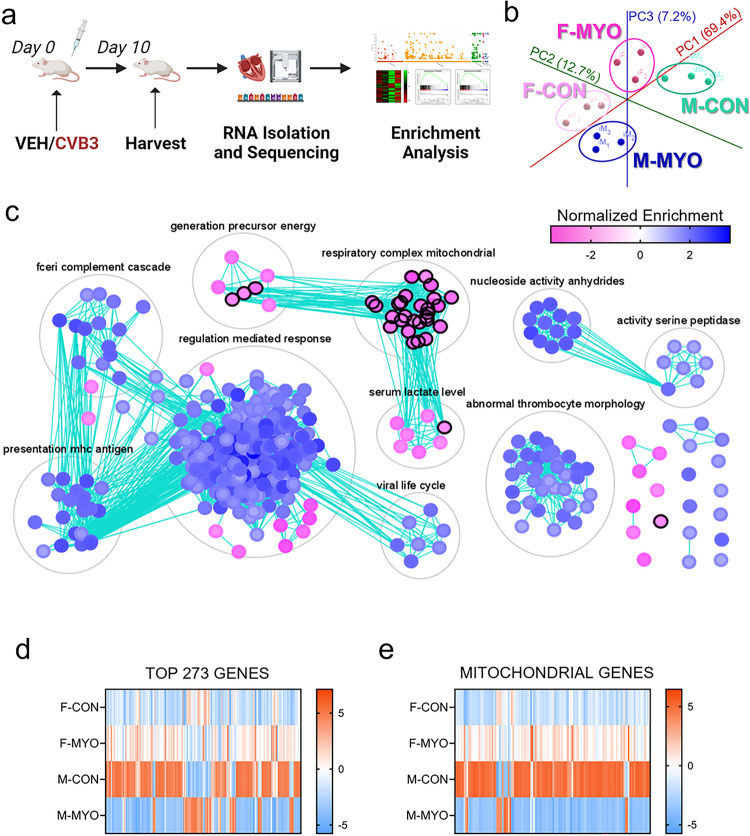
RNA sequencing reveals sex differences in immune and mitochondrial genes **a,** RNA-sequencing experimental pipeline; **b,** principal component analysis plot showing female controls (F-CON), females with myocarditis (F-MYO), male controls (M-CON), and males with myocarditis (M-MYO); **c,** results from GSEA pre-ranked plotted on Cytoscape with Enrichment Map and AutoAnnotate, pink = F-MYO and blue = M-MYO, nodes circled in black are mitochondrial-related pathways; heat map for **d,** the top 273 most differentially expressed genes and **e,** mitochondrial genes between all four groups.

We then examined sex differences in myocarditis using bulk-tissue RNAseq (an overview of the experimental design is illustrated in Figure 2a). PCA analysis revealed good separation between groups and high similarity within groups (Figure 2b). To better understand the mechanisms underlying sex differences in myocarditis, we performed gene set enrichment analysis (GSEA) of RNAseq data comparing males and females with myocarditis versus controls. When we compared females and males with myocarditis, we found males with myocarditis (blue) had significantly enriched clusters (i.e., auto-annotated grouped gene sets) for the following gene pathways compared to females: regulation mediated (immune) response, viral life cycle, presentation MHC antigen, Fc-receptor complement cascade, nucleoside activity anhydrides, abnormal thrombocyte morphology, and activity of serine peptidases (Figure 2c). Females with myocarditis (pink) had significantly enriched clusters for the following gene pathways compared to males: respiratory complex mitochondrial, generation precursor energy, and serum lactate levels (Figure 2c). Non-super-clustered gene sets (i.e., nodes) and their identities are displayed in **Additional File 1: Figure S2.** These data indicate that females with myocarditis have higher expression of mitochondrial respiratory transcripts than males. In contrast, males have higher expression of immune system genes compared to females, which is consistent with histology findings (Figure 1).

We used Cytoscape to generate heat maps from RNA sequencing data for the top 273 differentially expressed genes between males and females with myocarditis or controls (Figure 2d). These data revealed distinct gene profiles between each group with 200 of the top 273 genes being increased in females during myocarditis compared to males with myocarditis (Figure 2d). Females with myocarditis upregulated 216 genes compared to female controls in contrast to males with myocarditis that downregulated 210 genes compared to male controls (Figure 2d).

To better understand sex differences in mitochondrial genes, we selected mitochondrial gene sets in Cytoscape to generate a heat map containing 132 differentially expressed and mitochondrial-specific genes comparing females to males with myocarditis or controls (Figure 2e). The mitochondrial gene expression differences between controls and by sex were very similar to the findings of the top 273 genes. Females with myocarditis had higher expression of 118 of 132 mitochondrial genes compared to males with myocarditis (Figure 2e). Females upregulated 119 of 132 mitochondrial genes during myocarditis compared to female controls while males downregulated 120 mitochondrial genes compared to male controls (Figure 2e). These data demonstrate that males with myocarditis have decreased mitochondrial-related transcriptional support whereas females with myocarditis have increased mitochondrial-related transcript support during myocarditis.

**Figure 3:**
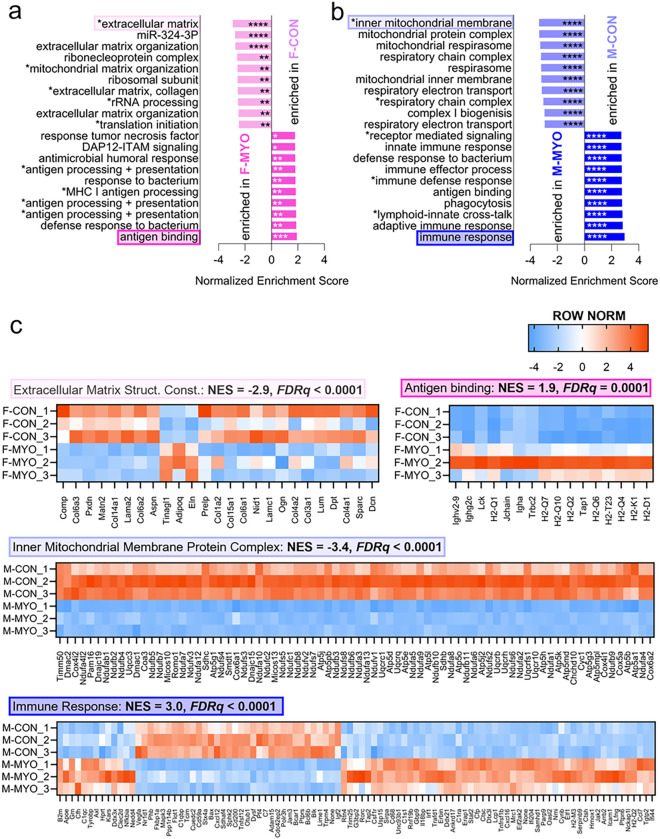
Sex-specific gene expression pathways during myocarditis The top ten most enriched pathways from GSEA ranked by normalized enrichment score comparing **a,** female controls (F-CON) to females with myocarditis (F-MYO), **b,** male controls (M-CON) to males with myocarditis (M-MYO) (pathway text marked with astricts indicate abbreviated pathway names, see supplement for abbreviations and full pathway names) **c,** Row normalized heatmaps for the most enriched pathways from F-CON vs F-MYO and M-CON vs M-MYO, respective to colors highlighting pathways in (a) and (b). NES = normalized enrichment score, Extracellular Matrix Structural (Struct.) Constituent (Const.). *FDRq<0.05,**FDRq<0.01, *** *FDRq*<0.001, **** *FDRq*<0.00001

To better understand gene pathways that differed by sex during myocarditis we plotted the top ten significant gene pathways from GSEA ranked by normalized enrichment score (NES) for control versus myocarditis (Figure 3a,b) and by sex (Figure 3c). We found that uninfected female hearts were enriched for gene sets related to mitochondrial and cellular homeostasis (Figure 3a). Increased expression of transcripts related to immune activation such as “antigen processing and interaction” were found in females during myocarditis (Figure 3a). Males with myocarditis transitioned from mitochondrial homeostasis in uninfected hearts to a proinflammatory immune response during myocarditis (Figure 3b). We generated heatmaps corresponding to highlighted top significantly enriched pathways in the female control versus female myocarditis comparison (extracellular matrix structural constituent and antigen binding, respectively) and for the male control versus male myocarditis comparison (inner mitochondrial membrane protein complex and immune response, respectively) which can be found with NES and false discovery rate (FDRq) values in Figure 3c.

**Figure 4:**
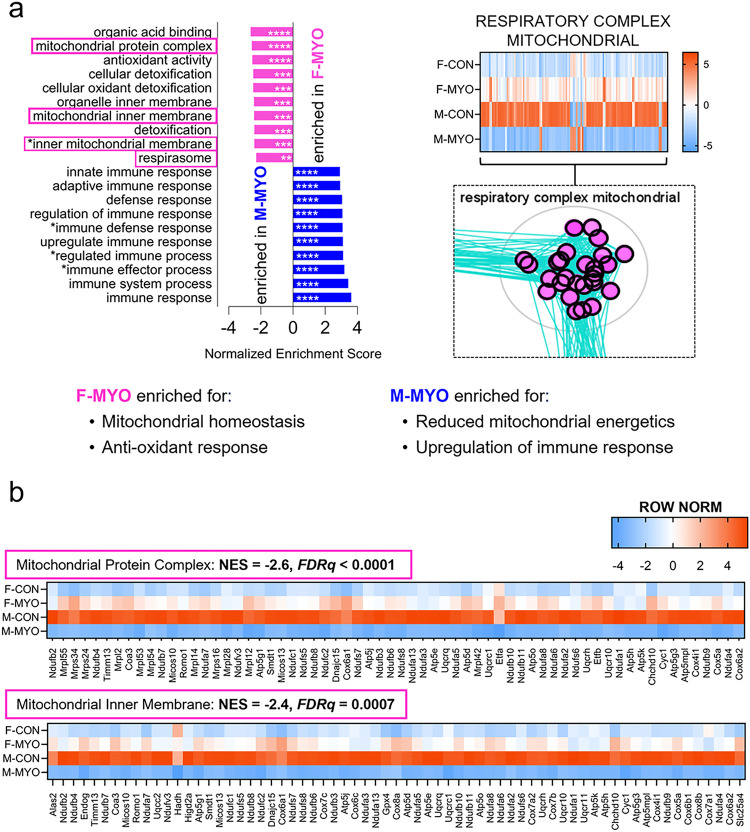
Sex differences in differential gene expression pathways during myocarditis The top ten most enriched pathways from GSEA ranked by normalized enrichment score comparing **a,** females with myocarditis (F-MYO) and males with myocarditis (M-MYO) and heatmap of auto-annotated cluster of pathways/nodes describing the mitochondrial respiratory complex, **b,** Row normalized heatmaps for pathways highlighted in pink for F-MYO (from F-MYO vs M-MYO comparison; other 2 pathways share common genes with those in (b) and are available in Supplemental Figure 7). *FDRq<0.05, ** *FDRq*<0.01, *** *FDRq*<0.001, **** *FDRq*<0.00001

A direct comparison of females to males with myocarditis revealed that females were enriched for pathways related to mitochondrial homeostasis and anti-oxidant responses while males were enriched for pathways related to the innate and adaptive immune responses (Figure 4a). We generated a heatmap showing all four groups of genes and gene sets in the super-cluster auto annotated as “respiratory complex mitochondrial”, which contained enriched gene sets related to the mitochondrial respiratory chain. Similar to the findings of the heatmap of all mitochondrial genes in Figure 2e, females had higher expression of 114 of 127 genes in this super-cluster (Figure 4a). Females with myocarditis upregulated 115 genes compared to female controls and males with myocarditis downregulated 116 genes compared to male controls (Figure 4a). We generated heatmaps and highlighted mitochondrial enriched gene sets, which were significantly more enriched in females with myocarditis compared to males with myocarditis including mitochondrial protein complex (NES = −2.6, *FDRq < 0.0001*) and mitochondrial inner membrane (NES = −2.4, *FDRq = 0.0007*) (Figure 4b). Inner mitochondrial membrane protein complex (NES = −2.4, *FDRq = 0.0007*) and respirasome (NES = −2.3, *FDRq = 0.004*) were also significantly enriched in females with myocarditis and mostly contained common genes with the pathways shown in Figure 4b; these heatmaps can be found in **Additional File 1: Figure S3.** Thus, aside from sex differences in immune changes during myocarditis, which have been well characterized in the past, the main difference in cardiac transcript enrichment between males and females with myocarditis occurred in pathways related to mitochondrial function.

**Figure 5:**
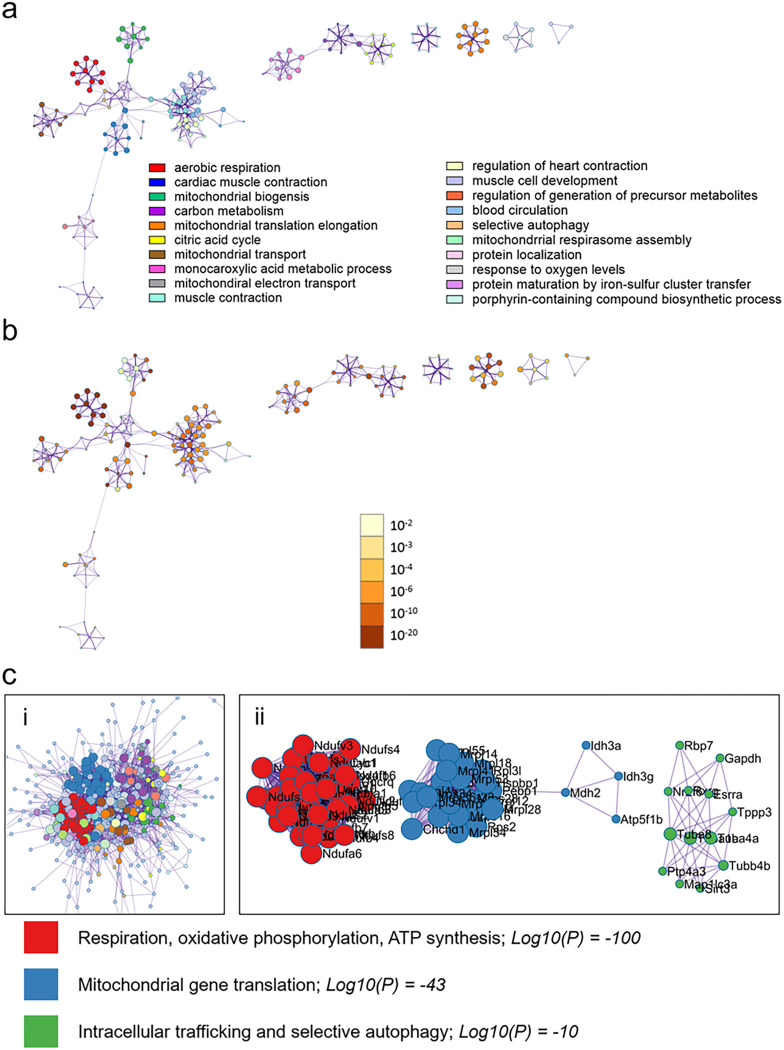
Females with myocarditis are enriched for pathways related to mitochondrial respiration compared to males with myocarditis Metascape enrichment results for females with myocarditis (comparing females and males with myocarditis) **a,** the top enriched pathways colored by cluster/pathway and **b,** by p-value. Top enriched pathways **c,** Protein-protein interaction analysis clustered by interaction outside of pathways (i) and interaction inside pathways (ii). Log10(P) vals are derived by averaging the Log10(P) vals for the 3 MCODE annotations, rounded to whole number with colors indicating respective pathways. Images in (i) are cropped to show the bulk of pathways and interactors and the top 3 pathways only are shown in (ii).

**Figure 6:**
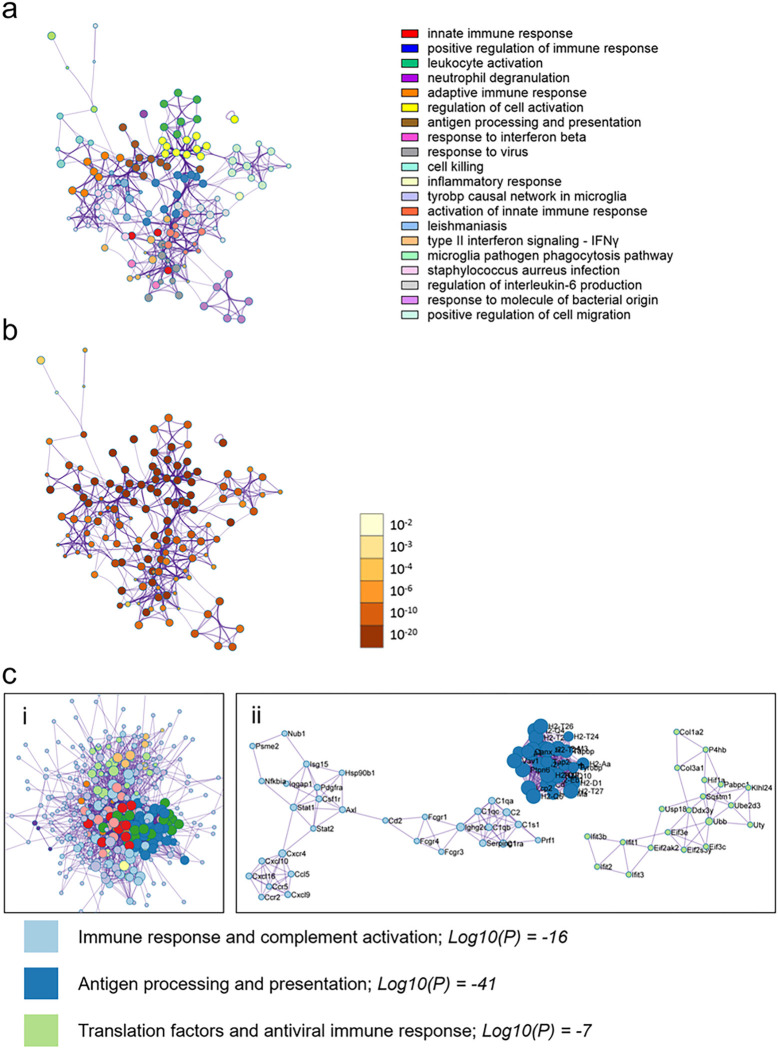
Males with myocarditis are enriched for pathways related immune activation compared to females with myocarditis Metascape enrichment results for males with myocarditis (comparing females and males with myocarditis) **a,** the top enriched pathways colored by cluster/pathway and **b,** by p-value. Top enriched pathways **c,** Protein-protein interaction analysis clustered by interaction outside of pathways (i) and interaction inside pathways (ii). Log10(P) vals are derived by averaging the Log10(P) vals for the 3 MCODE annotations, rounded to whole number with colors indicating respective pathways. Images in (i) are cropped to show the bulk of pathways and interactors and the top 3 pathways only are shown in (ii).

To ensure that the observed sex differences in mitochondrial transcriptional enrichment were not a result of the enrichment method performed (i.e., GSEA Pre-Ranked), we additionally performed enrichment analysis using Metascape [17]. Metascape enrichment for females with myocarditis compared to controls versus males with myocarditis compared to controls were broadly similar to GSEA enrichment findings in Figure 2 and can be found in **Additional File 1: Figures S4–7**. When we directly compared females with myocarditis to males with myocarditis, we confirmed that females with myocarditis were significantly enriched for pathways supporting mitochondria homeostasis and cell energetics (Figure 5) and males with myocarditis were enriched for pathways related to upregulation of the immune response (Figure 6). Importantly, MCODE_1 clustering of protein-protein interaction analysis by pathway, comprised of Reactome.org enrichment terms for respiratory electron transport (R-MMU-611105, and R-MMU-163200) and Gene Ontology oxidative phosphorylation (GO:0006119) pathways, were all enriched at Log10p values of −100 further highlighting that mitochondrial pathways were the primary enrichment signature in females with myocarditis compared to males with myocarditis (Figure 5c).

**Figure 7:**
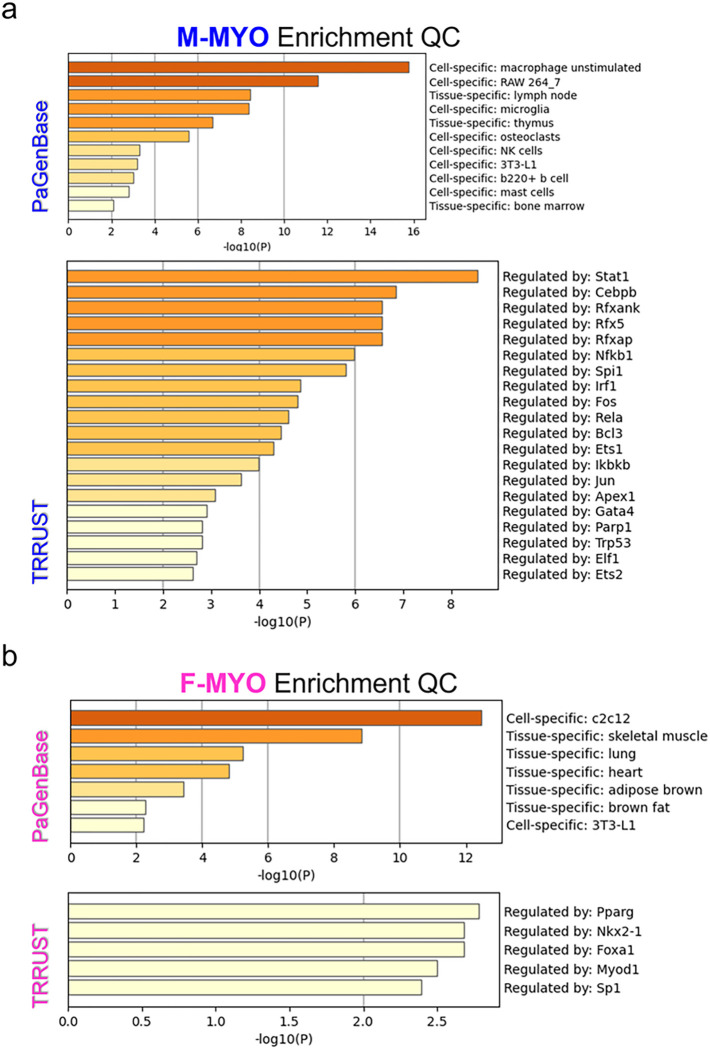
Metascape Enrichment QC shows cell specific signals and identifies potential transcriptional regulators of sex differences in myocarditis **a,** Enrichment quality control (QC) for males with myocarditis (M-MYO) shows cell specific enrichment signals from Pattern Gene Database (PaGenBase) and suggested transcription factors from Transcriptional Regulatory Relationships Unraveled by Sentence-based Text mining (TRRUST) and for **b,** females with myocarditis (F-MYO)

Enrichment quality control metrics from Metascape revealed cell-specific signatures for males with myocarditis compared to females with myocarditis which mirrored the known prevalence of immune cells in the heart with highest to lowest being macrophages, T cells, natural killer (NK cells), B cells and mast cells (Figure 7a) [16]. The most enriched transcription factor in males with myocarditis was signal transducer and activator of transcription (STAT)1, which is known to mount interferon (IFN) and T helper (Th)1/M1 immune responses that clear CVB3 infection during myocarditis [18, 19] (Figure 7a). In contrast, females with myocarditis were more enriched for myoblasts (c2c12), myocytes and other cardiac cell components with an absence of immune cells compared to males with myocarditis (Figure 7b). In contrast, the top enriched transcriptional regulator in females with myocarditis was peroxisome proliferator-activated receptor gamma (PParg), a transcriptional regulator associated with metabolic shift to integrate glycolysis and lipid anabolism in the failing heart [20].

In **Table S1**, we display the top 5 most enriched gene sets from gProfiler comparing females and males with myocarditis, which were primarily comprised of gene sets related to mitochondrial homeostasis. The top enriched pathways from gProfiler for each source were: electron transfer activity (Gene Ontology (GO): Molecular Functions/MF), electron transport chain (GO: Biological Process/BP), mitochondrial inner membrane (GO: Cellular Component/CC), oxidative phosphorylation (Kyoto Encyclopedia of Genes and Genomes/ KEGG), and the citric acid cycle and respiratory electron transport (Reactome/Reactome.org) (**Additional File 1: Table S1**). Overall, these results indicate that females with myocarditis have a stronger mitochondrial gene signature compared to males.

### Males with myocarditis have lower expression of electron transport chain genes compared to females

**Figure 8:**
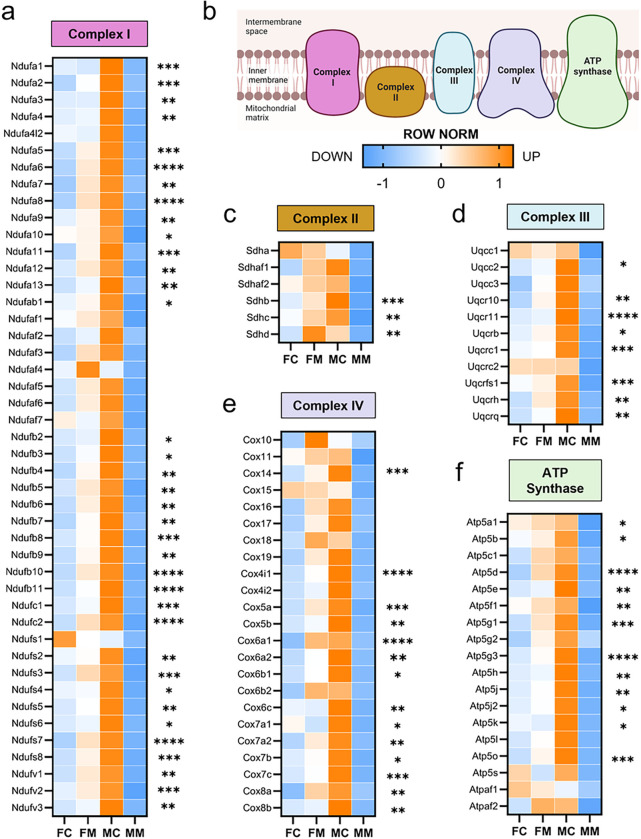
Sex differences in mitochondrial electron transport genes during myocarditis Row normalized RPKM comparing female controls (FC), females with myocarditis (FM), male controls (MC) and males with myocarditis (MM) for nuclear encoded genes for **a,** complex I, **b,** color-coded illustration of the mitochondrial electron transport chain; **c,** complex II, **d,** complex III, **e,** complex IV, and **f,** ATP synthase. *p<0.05, ** *p*<0.01, *** *p*<0.001,****p<0.0001

Based on our finding of sex differences in pathways related to mitochondrial respiration during myocarditis, we next focused our analysis on mitochondrial electron transport chain (ETC) genes (Figure 8). Using reads per kilobase per million (RPKM) from RNAseq results, we compared the expression of murine nuclear encoded ETC transcripts for each complex in the ETC. We found that 36 of 45 genes (80%) that form Complex I were significantly lower in males with myocarditis compared to females with myocarditis (Figure 8a,b), suggesting Complex I dysfunction in males with myocarditis. Significant differences in genes comparing males to females with myocarditis are indicated by asterisks (Figure 8). Males with myocarditis also had significant decreases in 3 genes out of 6 (50%) in Complex II (Figure 8c), 8 genes out of 11 (73%) in Complex III (Figure 8d), 14 genes out of 23 (61%) in Complex IV (Figure 8e), and 12 genes out of 18 (67%) in the ATP synthase compared to females with myocarditis (Figure 8f). These findings show that expression of nuclear encoded mitochondrial respiratory chain transcripts increase in females during myocarditis whereas they decrease in males.

### ERRa identified using TRANSFAC as a candidate transcription factor that may regulate mitochondrial genes

**Figure 9:**
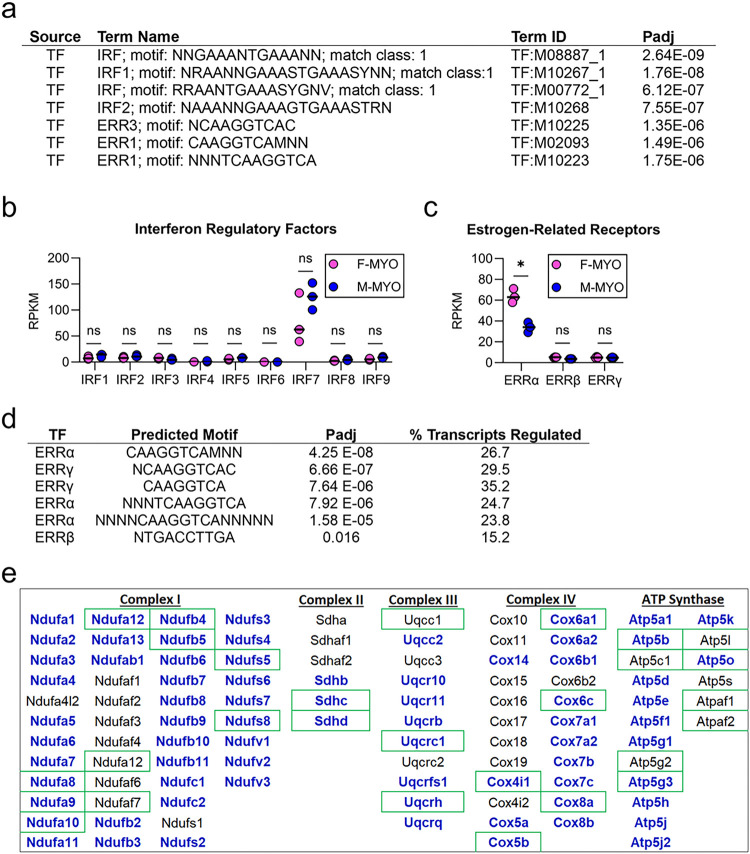
TRANSFAC analysis identifies interferon regulatory factors and estrogen-related receptors as potential mediators of sex difference during myocarditis **a,** TRANSFAC results comparing females with myocarditis (F-MYO) and males with myocarditis (M-MYO); RPKM (reads per kilobase per million) using false discovery rate to compare F-MYO and M-MYO for **b,** interferon regulatory factors (IRFs) and **c,** estrogen-related receptors (ERRs); **d,** predicted binding capacity of ERRs for electron transport chain transcripts; **e,** significantly different transcripts by sex in electron transport genes are indicated by bold blue lettering, green boxes indicate genes that ERRa predicted to bind to.

To identify transcriptional regulators that might globally affect the major changes in cellular mitochondrial energetic pathways according to sex that we observed, we used the gProfiler “*TRANScription FACtor database*” (TRANSFAC) to identify candidate transcription factors. TRANSFAC analysis identified interferon regulatory factors (IRFs) and estrogen-related receptors (ERRs) as the top potential regulators of gene differences between males and females with myocarditis (Figure 9a). We compared expression of all nine IRFs (Figure 9b) and all three ERRs (Figure 9c) from RNA sequencing data and found that none of the nine IRFs were significantly different by sex but ERRα was significantly higher in females with myocarditis compared to males (FDR = 0.03).

We assessed the predicted binding capacity of ERRs among the nuclear encoded mitochondrial respiratory chain complexes examined in Figure 8 using TRANSFAC. ERRα and ERRγ shared a core predicted binding motif of TCAAGGTCA with ERRα present in the proximal promoter of around 30% of the nuclear encoded mitochondrial respiratory chain complex transcripts (Figure 9d). This is in line with a previous study that showed that both ERRa and ERRg target a common set of promoters involved in mitochondrial respiration and ATP production in the hearts of male mice [21]. Indeed, respiratory chain complex genes that were predicted to be bound by ERRa (or ERRg) are indicated by green boxes and those that were significantly different by sex in Figure 8 are indicated with bold blue lettering in Figure 9e. These findings suggest that ERRs may influence the sex differences in mitochondrial gene expression that were observed during myocarditis.

### Females upregulate master regulators of mitochondrial homeostasis during myocarditis

**Figure 10:**
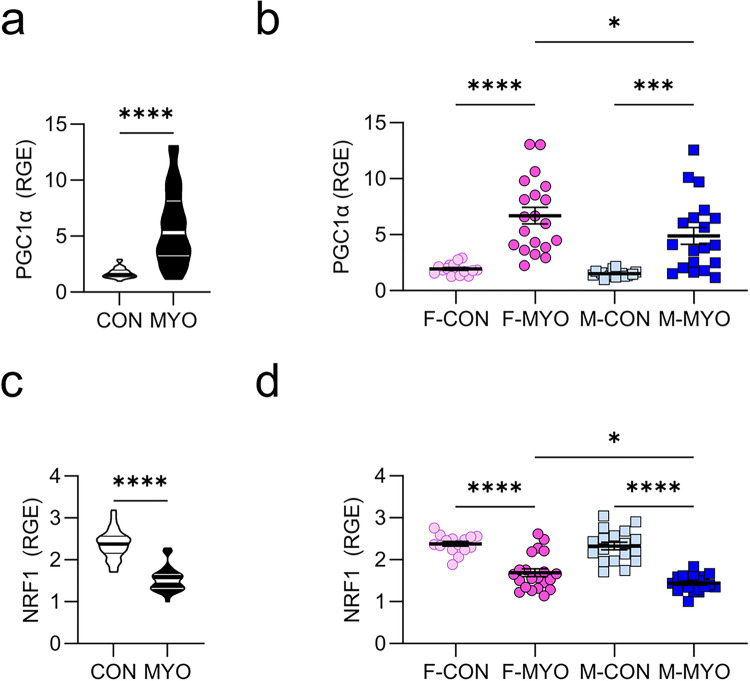
Females with myocarditis express higher levels of mitochondrial master regulators PGC1a and NRF1 Relative gene expression (RGE) for controls (CON, *n* = 33–35) versus mice with myocarditis (MYO, *n* = 35–39) and for F-CON (*n* = 15), F-MYO (*n* = 20–21), M-CON (*n* = 17–18), and M-MYO *n* = 19–20) for **a-b,** PGC1a; and **c,d,** NRF1.

Because we found sex differences in the expression of mitochondrial ETC genes (Figure 8), we examined whether sex differences existed in global regulators of mitochondrial metabolism including PGC1α and nuclear respiratory factor 1 (NRF1). Using qRT-PCR, we found that PGC1a levels were significantly increased in the heart during myocarditis when males and females with myocarditis were combined compared to controls (*p* < 0.0001) (Figure 10a) or examined individually compared to controls (females *p* < 0.0001, males *p* = 0.0003) (Figure 10b). Comparing males to females with myocarditis, females with myocarditis had significantly higher levels of PGC1a in the heart compared to males (*p* = 0.0458) (Figure 10b).

PGC1α interacts with NRF1 leading to transcription of mitochondrial genes including ATP synthase, cytochrome-c, cytochrome-c-oxidase subunit IV, and mitochondrial transcription factor A which activates mitochondrial DNA replication and transcription [22–24]. We found that NRF1 RNA levels were significantly decreased when males and females with myocarditis were combined compared to controls (*p* < 0.0001) (Figure 10c). This was also observed when NRF1 levels were examined in males with myocarditis versus controls (*p* < 0.0001) or females with myocarditis versus controls (*p* < 0.0001) (Figure 10d). However, NRF1 levels in the heart during myocarditis were significantly higher in females compared to males (*p* = 0.0315) (Figure 10d).

**Figure 11:**
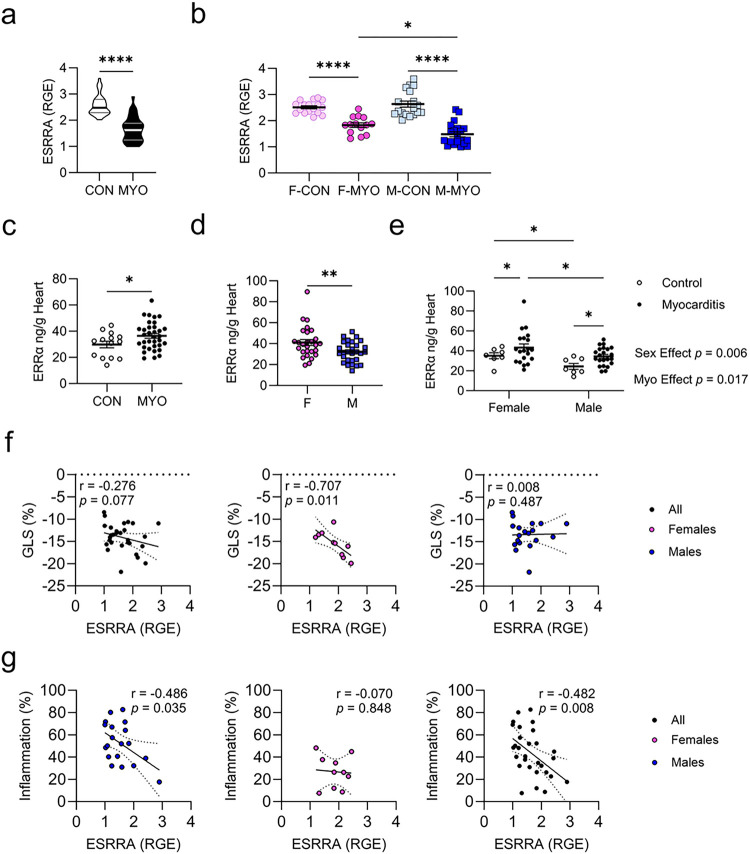
Females with myocarditis express higher levels of ERRαcompared to males Relative gene expression (RGE) for controls (CON, *n* = 33–35) versus mice with myocarditis (MYO, *n* = 35–39) and for F-CON (*n* = 15), F-MYO (*n* = 20–21), M-CON (*n* = 17–18), and M-MYO *n* = 19–20) for **a,b,** ERRa ELISA of ERRa protein from whole heart homogenate supernatant comparing **c,** CON (*n* = 14) to MYO (n= 42); **d,** all females (F, *n* = 27) to all males (M, *n* = 29) regardless of disease state; **e,** two-way ANOVA F-CON (*n* = 7), F-MYO (*n* = 20), M-CON (*n* = 7), and M-MYO (*n* = 22). Two-tailed assessment of correlations for, **f** ERRα gene expression and global longitudinal strain (GLS (%)), and **g** for ERRα gene expression and myocarditis severity (Inflammation %) scored by H+E stain.

We also assessed ERRa (ESRRA mRNA) by sex, which had been identified using TRANSFAC (Figure 9). ERRa is designated as an orphan nuclear receptor [25–27] but recent evidence suggests its endogenous ligand may be cholesterol [28–30]. ERRα displays some basal activity during nominal cell states but transcriptional activity is enhanced by co-activator interaction with PGC1α [24]. PGC1a is a transcriptional co-activator protein that binds ERRa (not as a ligand but as a co-factor [31]) and promotes its transcriptional activity [25]. ERRa-PGC1a have been found to regulate hundreds of genes involved in mitochondrial oxidative phosphorylation, the tricarboxylic acid (TCA) cycle, fatty acid beta-oxidation, and glucose and lipid metabolism [25, 26, 32–35]. When we examined ESRRA mRNA levels by qRT-PCR during myocarditis, we found that ESRRA was significantly decreased in mice with myocarditis compared to controls (*p* < 0.0001) (Figure 11a). This was also true when we examined females with myocarditis compared to controls (*p* < 0.0001) or males with myocarditis were compared to controls (*p* < 0.0001) (Figure 11b). Similar to PGC1a and NRF1, we found that females had significantly higher levels of ESRRA during myocarditis compared to males (*p* = 0.0128) (Figure 11b).

To further investigate ERRa levels during myocarditis we examined heart protein levels of ERRα by ELISA. At the protein level, we found that ERRα was significantly increased when males and females with myocarditis were combined compared to controls (*p* = 0.0486) (Figure 11c) and in all females compared to all males regardless of disease status (*p* = 0.0094) (Figure 11d). To determine the effect of sex versus myocarditis in ERRα protein expression, we performed two-way ANOVA and found a significant effect of sex (*p* = 0.006) and myocarditis (*p* = 0.017), indicating sex differences drive the main effect (Figure 11e). We also found that ERRa protein levels were significantly increased in females with myocarditis compared to controls (*p* = 0.0340) and in males with myocarditis compared to controls (*p* = 0.0340) (Figure 11e). Importantly, ERRa protein levels were significantly increased in females with myocarditis compared to males with myocarditis (*p* = 0.0234) (Figure 11e). Interestingly, ERRa protein was also significantly increased in females without myocarditis compared to males without myocarditis (*p* = 0.0234) (Figure 11e). Thus overall, ERRα protein levels were elevated in the heart of females compared to males during myocarditis.

**Figure 12:**
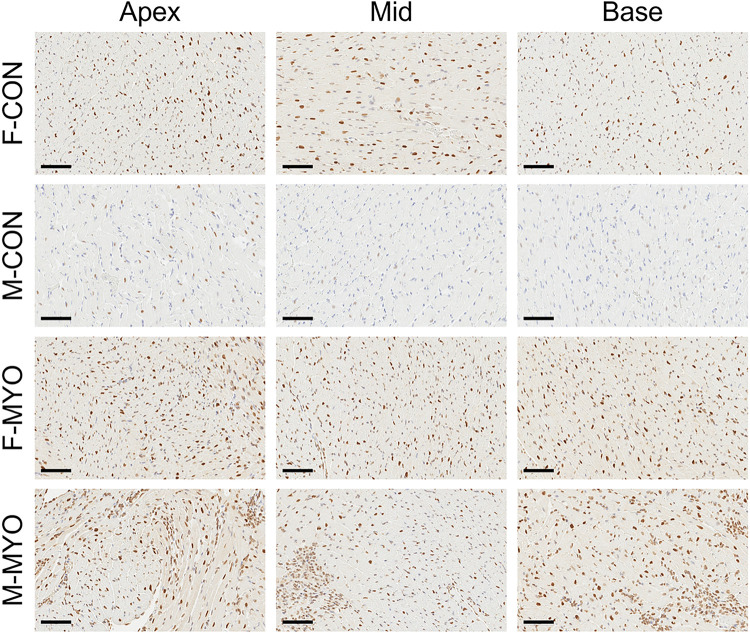
In situ expression of ERRα demonstrates sex differences Representative heart sections (based on H&E scores) for F-CON, M-CON, F-MYO, and M-MYO stained for ERRα. Images of the myocardium were taken at the base, middle (mid) and apex of the heart for each sample. Sale bars = 70 μm.

To examine cardiac expression of ERRα in the murine heart, we selected representative slides based on average inflammation scores and performed immunohistochemistry (IHC). We selected representative images at the base, mid, and apical myocardium for each heart section. We observed higher ERRa staining intensity in the female control hearts compared to male controls where staining appeared to be most concentrated in cardiomyocyte nuclei (Figure 12). Increased staining intensity was also found for ERRα in females compared to males with myocarditis (Figure 12). ERRα expression was observed for individual immune cells and inflammatory foci in males and females as well as cardiomyocyte nuclei during myocarditis (Figure 12).

To better understand function and disease relevance of ESRRA expression in the context of myocarditis, we performed two-tailed correlation analysis comparing ESRRA transcript levels and global longitudinal strain (GLS) obtained using echocardiography (Figure 11f) and ESRRA transcript levels and cardiac inflammation (scored histologically) (Figure 11g). We did not find a significant correlation when assessing ESRRA transcript levels to GLS when we included males and females with myocarditis together (*p* = 0.077) but found a significant correlation in females (*p* = 0.011) but not males (*p* = 0.487) (Figure 11f). In contrast, ESRRA transcript levels negatively correlated with cardiac inflammation when including males and females in the comparison (*p* = 0.008), but this effect was only significant in males (*p* = 0.035) and not females (*p* = 0.848) with myocarditis (Figure 11g). These findings indicate that ESRRA levels directly impact inflammation and cardiac function during myocarditis in a sex-specific manner.

## Discussion

In this study we show for the first time that male mice with CVB3 myocarditis have reduced mitochondrial transcription compared to females using an autoimmune model of CVB3 myocarditis that is highly translational to human disease [36]. We show that females with myocarditis have higher expression of several master regulators of mitochondrial homeostasis including PGC1a, NRF1 and ERRa compared to males. Females with CVB3 myocarditis had transcriptional evidence of better mitochondrial function and significantly less myocardial inflammation than males. A sex-specific effect of ERRa on inflammation and cardiac function suggests a potential regulatory mechanism for our observed sex differences in mitochondrial gene transcription.

PGC1a was originally identified as a regulator of mitochondrial function in brown adipose tissue, but was later also found to be expressed at high levels in cardiac tissue where it influences cardiovascular health and disease [25, 37]. PGC1a globally regulates mitochondrial pathways in response to stresses such as cold, fasting and infection [35, 38–41]. Thus, the metabolic stress of CVB3 infection is a likely explanation for the elevated levels of PGC1a that we observed in males and females with myocarditis compared to controls (Fig. 10a,b). Additionally, a study by Dufour et al. using ERRα deficient mice, found that when ERRa was low in the heart PGC1a was elevated as a compensation mechanism [21].

ERRa has been found to be critical in regulating mitochondrial homeostasis in the heart demonstrated by Dufour et al. using male ERRa deficient (KO) C57BL/6 mice [21]. They found that ERRa targeted mitochondrial NRF1, cyclic AMP-response element binding protein (CREB), and STAT3 [42]. Surprisingly, we observed an inverse relationship between mRNA and protein levels of ERRα in the heart with increased levels by ELISA and IHC in males and females with myocarditis. Regardless of mRNA levels, ERRα protein levels can be highly regulated by post-translational modifications and metabolic stress [43]. Previously, it was notably shown that under specific cellular metabolic stress conditions, such as reactive oxygen species (ROS) exposure, ERRα protein levels can be dramatically altered in a proteasome-dependent manner [43]. Similarly, insulin or glucose stimulation increased ERRα protein levels without altering mRNA expression in hepatocytes [44], further strengthening the hypothesis that ERRα protein levels can be altered under metabolic pressure independently of gene expression. Indeed, transcription factor expression often does not necessarily provide detailed information as to the direct actions of that transcription factor; and in this case, how ERRα activity may differ by sex during myocarditis. Future studies utilizing methods to characterize the genomic interactions of ERRα would be useful in elucidating sex-specific transcriptional activity.

Interleukin (IL)-1a, IL-1b, and tumor necrosis factor (TNF)a are known to activate the transcriptional activity of PGC1a through direct phosphorylation of p38 mitogen-activated protein (MAP) kinase [24, 45]. We found previously that IL-1b levels are increased in the heart of males with myocarditis, while cardiac levels of TNFa are increased in females in our CVB3 model of myocarditis [10, 46]. Most cardiac inflammatory cells during acute myocarditis at day 10 pi are CD11b+ (macrophages and mast cells) that express TLR4 and release IL-1b [16]. We showed previously that elevated IL-1b levels in the heart directly correlate to elevated cardiac inflammation in males with myocarditis and poor cardiac function by echocardiography [10]. Importantly, Remels et al. showed that elevated TNFa levels in cardiomyocytes in culture following CVB3 infection were directly associated with decreased PGC1a mRNA levels [47].

Additional evidence of the negative effect that IL-1b can have on mitochondrial gene expression in males was found in studies by Ge et al. [19, 48, 49]. Calpain is a calcium-dependent protease that facilitates apoptotic signaling and localizes to the mitochondria during CVB3 infection to proteolyze mitochondrial substrates, leading to increased mitochondrial fission (mitochondrial fragmentation due to pathological or physiological stress). Inhibition of calpain reduced mitochondrial fission and cardiomyocyte apoptosis during myocarditis [48]. Liu et al. showed that mitochondrial calpain-1 induces mitochondrial dysfunction and ROS production which activated the NLRP3 inflammasome, which leads to IL-1b production [49]. Macrophages, which are the predominant infiltrating immune cells during myocarditis, were found to respond to CVB3 infection by upregulation of calpain-4; RNA sequencing of CVB3 infected macrophages in vitro revealed predominant enrichment for pathways related to macrophage maturation and interleukin signaling, and loss of calpain-4 reduced IL-1b expression [19]. Although we did not specifically examine IL-1b in this study, our previous findings may be relevant to the current results that suggest that elevated inflammatory cells and cytokines, especially IL-1b, in the heart of males during acute CVB3 myocarditis [10] may directly contribute to lower PGC1a levels in males than females leading to decreased mitochondrial gene expression in the heart at that timepoint.

In general, sex differences are known to exist in mitochondrial bioenergetics [50, 51], but we provide sex-specific information in the context of viral myocarditis. Similar to previous studies that examined gene changes in the heart during CVB3 myocarditis in male mice [10, 47, 52], we found that the predominant gene expression changes aside from immune pathways were mitochondrial genes. Previously, Remels et al. reported that PGC1a mRNA and NRF1 protein levels were significantly decreased in the heart of male mice with CVB3 myocarditis compared to controls from day 4 to 7 pi [47]. They also found decreased gene expression profiles for ETC genes during myocarditis in males [47], similar to our results, but they did not examine females with myocarditis.

Ebermann et al. also examined gene expression in males with CVB3 myocarditis comparing C57BL/6 (B6) to A.SW/SnJ mice [53]. They used a tissue culture CVB3-induced model that produces similar inflammation in these two strains of mice but different cytokine profiles [53]. This tissue-culture CVB3 model produces a completely different myocardial immune profile than our model of autoimmune CVB3-myocarditis comparing BALB/c to B6 mice [9, 54, 55]. However, Ebermann et al. found that A.Sw/SnJ male mice with myocarditis have significantly lower ETC gene expression compared to controls that was directly related to the level of viral replication in the heart [53]. In our model of CVB3 myocarditis there are no sex differences in VP1 RNA levels or viral replication based on plaque assay during acute myocarditis [16]. The findings of Ebermann et al. may reflect, however, the finding of Sin et al. who showed in cultured cardiomyocytes that CVB3 localizes to mitochondria, induces mitophagy, and disseminates from the cell in an extracellular autophagosome-bound virus-laden mitochondrial complex [56]. Sin et al. showed that upstream suppression of the mitophagy pathway in HL-1 cardiomyocytes using small interfering RNA (siRNA) targeted to dynamin-related protien-1 (DRP1) or mitochondrial division inhibitor (Mdivi-1) significantly reduced virus production from cardiomyocytes [56] (as mitochondrial fission is an early stage of mitophagy). Other viruses that cause myocarditis such as human immunodeficiency virus (HIV), hepatitis B and C, influenza, Epstein-Barr virus and SARS-CoV-2 have been found to localize to mitochondria and hijack aspects of the mitochondrial machinery for replicationn [5, 57–59]. This might explain why so many diverse viruses without specific tropism for cardiac tissue (i.e., murine cytomegalovirus/ MCMV, SARS-CoV-2, CVB3) are able to cause myocarditis, since they can target a mitochondria-rich environment for replicatory advantage.

TRANSFAC analysis identified IRFs and ERRs as key transcription factors that could mediate sex differences in gene expression in our model. CVB3 infection strongly activates type I interferons (IFNas and IFNb) and type II (IFNg) IFN production during myocarditis to reduce viral replication via Toll-like receptor (TLR) activation including TLR3, TLR4, TLR7 and TLR9 and the transcription factor TIR domain-containing adaptor inducing interferon-β (TRIF) which is downstream of TLR3 and TLR4 [10, 46, 60–62]. Although IFNg is increased in our model of CVB3 myocarditis in males [16, 18], we showed that elevated IFN levels in male BALB/c mice with myocarditis are mediated by IL-18, which is downstream from TLR4, rather than traditional STAT4/IL-12 transcriptional activity [18, 63]. We do not observe a sex difference in viral levels in the heart during myocarditis in our CVB3 mouse model and sex differences in IFNg are not mediated by classic IFN signaling. Therefore, is not surprising that we did not observe a significant difference by sex of the nine IFN transcription factors (Fig. 5b).

Sex hormones are known to strongly drive the innate and adaptive immune response to infections in general and during myocarditis [8, 64, 65], and to confer sex differences in mitochondrial morphology and function via estrogen receptor (ER) nuclear and mitochondrial transcription factor activity [39, 66, 67]. The heart of females is known to have greater mitochondrial efficiency, fatty acid utilization during exercise, and calcium retention whereas males have more mitochondrial content, reactive oxygen species production, and higher calcium uptake rate for example [39, 66]. A summary of these known sex differences in mitochondrial-related genes and pathways can be found in **Additional File 1: Table S2.** ERRα was originally named based on its sequence homology to ERα [68]. Although sex differences in some mitochondrial gene expression pathways during CVB3 myocarditis may be explained by sex hormones, specifically estrogen via ERs, 17b-estradiol and other natural estrogens are not endogenous ligands for ERRα [68, 69]. Two groups have provided evidence that support the hypothesis that cholesterol is the endogenous ligand for ERRα with *in vitro* and *in vivo* data [28–30]. During nominal cellular states and unbound by its ligand, ERRα displays some transcriptional activity [25, 32, 70]. Based on structural homology, ERRs are speculated to share target genes, coregulatory proteins, and sites of action with ERs and therefore actively influence the estrogenic response [71]. The genotype-tissue expression (GTEx) project identified ERRα as a “sex-biased” transcriptional regulator in humans [72]. Overall, this could explain sex differences in ERRa expression.

Lee et al. found sex differences in ERRa levels in the brains of 4-week-old immature female but not male mice that had been treated with a chemical known to reduce mitochondrial function [73], suggesting sex differences in ERRa function prior to the production of circulating hormone production. De Jesus-Cortez et al. found that ERRa deficient adult female mice had defects in neural function in a mouse model of eating disorders, which mainly affect women, which was not observed in ERRa deficient male mice, and they concluded that ERRa was required for optimal mitochondrial function in females [74]. Watson et al. found sex-specific effects of ERRa expression in the hearts of female but not male mice in a model of heart failure [75]. We are the first to report sex differences in ERRα expression in the hearts of healthy mice and mice with viral myocarditis. Subsequent studies are needed to further characterize sex-specific effects of ERRα on mitochondrial function during CVB3 myocarditis. However, to fully characterize the sex differences in ERRa effects on gene regulation in healthy and mice with myocarditis, an analysis of gene-specific transcription factor (TF)-DNA interaction of ERRa is needed using chromatin immunoprecipitation (ChIP) or similar methods.

## Conclusion

In this study we show for the first time that males with CVB3 myocarditis have reduced mitochondrial gene expression of nuclear-encoded electron transport chain genes compared to females. Females had higher levels of global regulators of mitochondrial function compared to males which may promote mitochondrial homeostasis that protects females from cardiac damage following infection and inflammation. Future studies should characterize the direct transcriptional activity of the sex-differentially expressed orphan nuclear receptor ERRα.

## Methods

### Myocarditis Model

Male and female 6–8 week-old BALB/cJ mice (stock# 651) were obtained from Jackson Laboratory (Bar Harbor, ME). Mice were inoculated with sterile phosphate buffered saline (PBS) (vehicle control) or 10^3^ plaque forming units (PFU) of heart-passaged CVB3 intraperitoneally (ip) on day 0 and hearts collected on day 10 pi, as previously described [76]. This is an autoimmune model of myocarditis using live virus as the adjuvant that closely resembles experimental autoimmune myocarditis and human disease (reviewed in [54, 55]). The Nancy strain of CVB3 was originally obtained from the American Type Culture Collection (ATCC; Manassas, VA) and grown in Vero cells (ATCC), to create a tissue culture-derived virus stock as previously described [76]. Briefly, 100mL of tissue culture virus (10^3^ PFU) was injected ip into 4-week-old female BALB/c mice and virus obtained from hearts at day 3 pi by homogenization in Gibco Minimum Essential Media (Thermo-Scientific, Waltham, MA, 11095–080) supplemented with 2% heat inactivated FBS. Homogenized hearts were centrifuged at 4C for 20 min at 795g. Homogenized supernatant that contains infectious virus and damaged heart proteins (heart-passaged virus) was stored at −80 until used to induce myocarditis, as described in [76].

### Histology

Mouse hearts were cut longitudinally and fixed in 10% phosphate-buffered formalin and embedded in paraffin for histological analysis. 5 μm sections were stained with hematoxylin and eosin (H&E) to detect inflammation. Myocarditis was assessed as the percentage of the heart with inflammation compared to the overall size of the heart section using a microscope eyepiece grid, as previously [9, 77]. Sections were scored by two individuals blinded to the treatment group.

### Immunohistochemistry

Heart sections (5 mm) were stained with ERRa (ThermoFisher/Invitrogen, 1:1,000, cat**# PA5–28749**). An Envision+ anti-rabbit labeled polymer (K4003) and rat-on-rodent kit (RT517) (Biocare, Pacheco, CA) were used as secondary antibodies for rat antibodies. Stained slides were scanned using an Aperio AT2 slide scanner (Leica, Wetzlar, Germany). Representative fields of view from the apex, mid and base of ventricles were manually selected.

### Quantitative Real Time PCR

RNA was isolated from mouse hearts using Qiagen’s Fibrous Tissue Mini Kit (Qiagen 74704) before concentration (Abs. 260) and quality (Abs. 260/280) of preps was assessed using a Nanodrop. cDNA was generated using the iScript cDNA synthesis kit (Biorad, #1708891). Quantitative real time PCR (qRT-PCR) was assessed with Taqman probes (CD45/*Ptprc* Mm01293577_m1; CD11b/*Itgam* Mm00434455_m1; F4/80/*Adgre1* Mm00802529_m1; peroxisome proliferator-activated receptor gamma coactivator 1 (PGC1α)/*Ppargc1a* Mm01208835_m1; nuclear respiratory factor 1 (NRF1)/*Nrf1* Mm01135606_m1; estrogen-related receptor-a (ERRa)/*Esrra* Mm00433143_m1) and normalized against hypoxanthine phosphoribosyltransferase 1 (HPRT) (*Hprt*, Mm03024075_m1) to determine relative gene expression (RGE) using ΔΔCt as previously [77, 78].

### RNA Sequencing

At the time of harvest, half of the heart was collected for histological evaluation using H&E to determine the severity of myocardial inflammation and the other half was snap frozen in liquid nitrogen. Histology shown in Figure 1 is combined data from 3 separate experiments. The investigator selected 3 histologically representative samples of the overall dataset from a single experiment (see Figure S1; *n* = 3/group) and sent to the Mayo Clinic Genome Analysis Core for library preparation and bulk-tissue RNA sequencing. Libraries for this study were prepared using the core’s standard mRNAseq prep which uses poly A selection. RNA libraries were prepared using 200 ng of total RNA according to the manufacturer’s instructions for the TruSeq Stranded mRNA Sample Prep Kit (Illumina, San Diego, CA). The concentration and size distribution of the completed libraries was determined using an Agilent Bioanalyzer DNA 1000 chip (Santa Clara, CA) and Qubit fluorometry (Invitrogen, Carlsbad, CA). Libraries were sequenced at 50 million fragment reads per sample following Illumina’s standard protocol using the Illumina cBot and HiSeq 3000/4000 PE Cluster Kit. The flow cells were sequenced as 100 × 2 paired end reads on an Illumina HiSeq 4000 using HiSeq 3000/4000 sequencing kit and HiSeq Control Software HD 3.4.0.38 collection software. Base-calling was performed using Illumina’s RTA version 2.7.7

### RNA Sequencing Analysis

After next-generation RNA sequencing, the Mayo Clinic Genome Analysis Core provided differential expression data. We compared PBS control females vs. females with myocarditis, PBS control males vs. males with myocarditis, and females with myocarditis vs. males with myocarditis. Because of small group size, we assessed intra-group variability using *ClustVis* [79] by performing unsupervised hierarchical clustering using Euclidean row and column distances and principal component analysis (PCA).

Differential expression analysis was performed by the Mayo Clinic Genome Analysis Core and gene names were converted to murine ensemble IDs (ENSMUSG) for analysis. We performed enrichment analysis as in Reimand et al. [80]. For gProfiler, transcripts with nominal p-value < 0.05 were ordered from most to least significant and an ordered query was run (gene sets with 5–350 entities were included). At the time of analysis, we excluded duplicate transcripts or those not recognized by gProfiler. The same set of transcripts used for gProfiler were ordered by logFC to perform gene set enrichment analysis (GSEA) pre-ranked utilizing a combined gene matrix transposed (GMT) from gProfiler. GSEA pre-ranked was performed with default gene set size restriction (15–500) and permutation parameters (1,000).

GSEA results were plotted in Cytoscape (Version 3.7.23) using *Enrichment Map* with a node (i.e., gene set/pathway) cutoff of FDR(Q) value < 0.1 and edge cutoff of 0.375. Nodes were clustered based on shared genes and *AutoAnnotate* was used to identify clusters of nodes (sometimes referred to as “super-clusters” in this text). We selected all nodes to create a combined heat map of the top 273 genes and additionally selected mitochondrial-related nodes to create a combined heat map of the top 132 mitochondrial genes by group (row-normalized by Cytoscape). Combined heat maps for top NES pathways in F-CON vs F-MYO and M-CON vs M-MYO comparisons and mitochondrial pathways in F-MYO vs M-MYO comparison (including the combined auto-annotated cluster of gene sets “respiratory complex mitochondrial) were generated from Cytoscape (row-normalized). For Metascape enrichment analysis, we excluded all genes with *p* > 0.05, and ran corresponding gene sets for each phenotype with the *Express Analysis* option (Metascape.org)[17] for the following comparisons: F-CON vs F-MYO, M-CON vs M-MYO, and F-MYO vs M-MYO. We averaged MCODE clusters’ Log10p values (rounded to nearest whole number) to obtain values listed in Figures 5c and 6c.

A list of the murine nuclear encoded mitochondrial respiratory chain transcripts was generated from the Mouse Genome Informatics (MGI) database from Jackson Laboratories for respiratory chain (https://www.informatics.jax.org/go/term/GO:0005746) and the ATP synthase (https://www.informatics.jax.org/go/term/GO:0005753). Duplicates were removed and only transcripts of the mitochondrial respiratory chain were included. Transcripts and related data were used to create a combined gene expression matrix for each complex with row normalization (using the STANDARDIZE function in Excel). Transcripts not expressed across all four groups (PBS control females, PBS control males, myocarditis females, and myocarditis males) were excluded. The final list of transcripts was used to determine percent of transcripts predicted to be regulated via estrogen related receptors using TRANSFAC in gProfiler.

### ELISA

Frozen hearts were rapidly thawed and weighed to obtain tissue wet weight before homogenizing using a polytron homogenizer in minimum essential media (MEM) with 2% fetal bovine serum (FBS). Homogenized tissue was centrifuged at 3,000 rpm at 6°C for 20 minutes and the supernatant was collected for analysis. Whole heart ERRa protein expression was quantified using the Mouse Estrogen-Related Receptor Alpha ELISA Kit from MyBioSource (cat# MBS080310, San Diego, CA). Absorbance was used to calculate concentration relative to a standard curve and normalized to tissue wet weight, as previously [46, 77, 78, 81]. The lowest detection limit for the ERRa kit was 0.1ng/mL with a detection range of 0.25–8 ng/mL.

### Echocardiography

Cardiac function was determined by transthoracic echocardiography performed using the Vevo 3100 Ultrasound machine equipped with a MX550D 40MHz transducer mounted to a “3D Motor” (VisualSonics Inc., Toronto, Canada) Mice were sedated with 3% isoflurane, hair across the abdominal cavity was removed using Nair while isoflurane sedation was continued at 1–3% depending on animal heart rate, and ultrasonic transmission gel (Parker Laboratories, Fairfield, NJ) was applied to the thorax.^17,29,44^. A heart rate of 350–450 beats per minute (bpm) was maintained during the procedure. Two-dimensional (2D) parasternal long-axis (LAX) of the left-ventricle (LV) were acquired in B-mode. For echocardiography-derived global longitudinal strain (GLS), LAX images were analyzed using Vevo Strain analysis software (within Vevo LAB) with three cardiac cycles. Strain measures were derived from the formula for cardiac strain which is defined by the difference in movement of a wall from its starting position at end-diastolic diameter to its end position at end-systolic diameter divided by the original position of the wall. This effectively represents a percent-change in wall position composed of individual component vectors. For 2D LAX this includes the longitudinal and radial movement vectors.

### Statistical Analysis

Normally distributed data comparing two groups, determined with Prism, were analyzed using a 2-tailed Student’s t test. Multiple comparison analysis was performed by ANOVA with each group compared to the corresponding control group; 2-way ANOVA with repeated measures was used to determine the effect of sex vs. disease (myocarditis) using a main effects model. Multiple comparisons were performed with Holm-Sidak. Outlier analysis/exclusion was performed with ROUT (Q = 2%). Violin plots display mean and quartiles, other data are displayed as mean ± SEM. A value of *p* < 0.05 was considered significant. Adjusted p-values (from Prism) were used for multiple comparisons.

## Data Availability

The data that support the findings of this study are available from the corresponding author upon reasonable request. Data will be uploaded to an approved repository upon acceptance to journal.

## Highlights

- Viral myocarditis is more severe in males than females.
- ERRa and mitochondrial gene expression increased in females with myocarditis.
- ESRRA expression improves cardiac function in females with myocarditis.
- Low ESRRA is associated with increased myocarditis in males.
